# Synthesis and Enhanced Ethanol Gas Sensing Properties of the g-C_3_N_4_ Nanosheets-Decorated Tin Oxide Flower-Like Nanorods Composite

**DOI:** 10.3390/nano7100285

**Published:** 2017-09-22

**Authors:** Yan Wang, Jianliang Cao, Cong Qin, Bo Zhang, Guang Sun, Zhanying Zhang

**Affiliations:** 1 The Collaboration Innovation Center of Coal Safety Production of Henan Province, Jiaozuo 454000, China; yanwang@hpu.edu.cn; 2 State Key Laboratory Cultivation Bases Gas Geology and Gas Control (Henan Polytechnic University), Jiaozuo 454000, China; 3 School of Chemistry and Chemical Engineering, Henan Polytechnic University, Jiaozuo 454000, China; zhb@hpu.edu.cn (B.Z.); mcsunguang@hpu.edu.cn (G.S.); zhangzy@hpu.edu.cn (Z.Z.)

**Keywords:** nanocomposites, microstructure, gas sensor, flower-like SnO_2_ nanorod, graphitic carbon nitride

## Abstract

Flower-like SnO_2_/g-C_3_N_4_ nanocomposites were synthesized via a facile hydrothermal method by using SnCl_4_·5H_2_O and urea as the precursor. The structure and morphology of the as-synthesized samples were characterized by using the X-ray powder diffraction (XRD), electron microscopy (FESEM and TEM), and Fourier transform infrared spectrometer (FT-IR) techniques. SnO_2_ displays the unique 3D flower-like microstructure assembled with many uniform nanorods with the lengths and diameters of about 400–600 nm and 50–100 nm, respectively. For the SnO_2_/g-C_3_N_4_ composites, SnO_2_ flower-like nanorods were coupled by a lamellar structure 2D g-C_3_N_4_. Gas sensing performance test results indicated that the response of the sensor based on 7 wt. % 2D g-C_3_N_4_-decorated SnO_2_ composite to 500 ppm ethanol vapor was 150 at 340 °C, which was 3.5 times higher than that of the pure flower-like SnO_2_ nanorods-based sensor. The gas sensing mechanism of the g-C_3_N_4_nanosheets-decorated SnO_2_ flower-like nanorods was discussed in relation to the heterojunction structure between g-C_3_N_4_ and SnO_2_.

## 1. Introduction

As an n-type metal-oxide semiconductor, tin oxide (SnO_2_) has wide applications in many fields, such as lithium-ion batteries [1], photocatalysis [2], and gas sensors [3]. SnO_2_ has been investigated as a typical semiconductor gas sensor to ethanol because of its unique chemical properties and crystal structure [4]. As is known, the gas-sensing performance of SnO_2_-based sensors can be improved by means of morphology and size control. Hence, diverse shape-controlled SnO_2_ nanostructures have been synthesized, such as nanoflower [5], nanoarray [6], nanoplate [7], and nanowire [8]. These SnO_2_-based sensors exhibited good sensing properties, including low-cost and fast response and recovery.

However, there are some limitations which prevent the direct application of these sensors, such as poor electrical characteristics, high work temperature, and a low response [9]. Coupling SnO_2_ with other semiconductors to construct the heterojunction structure could be an efficient way to overcome these disadvantages. Therefore, many SnO_2_-based composites such as SnO_2_/r-GO [10,11,12,13,14,15], SnO_2_/ZnO [16,17,18,19], SnO_2_/Fe_2_O_3_ [20,21,22,23], and SnO_2_/NiO [24,25,26] have been synthesized as high-efficiency gas sensors. Graphitic carbon nitride (g-C_3_N_4_) is a two-dimensional (2D) semiconductor with a 2.7 eV band gap, which possesses good chemical stability and a large surface area. It is available to form an n/n junction structure with SnO_2_ [27]. For example, SnO_2_/g-C_3_N_4_nanocomposites with a strong heterojunction structure were designed and fabricated. The photocatalytic activity of the SnO_2_/g-C_3_N_4_ nanocomposites exhibited enhanced catalytic activity and stable cycle property [28]. Zhang et al. prepared the α-Fe_2_O_3_/g-C_3_N_4_ heterostructural nanocomposites as an ethanol gas sensor, and the composites exhibited a high response value (S = 7.76) to 100 ppm ethanol under a working temperature of 340 °C [29]. Zeng et al. successfully fabricated the α-Fe_2_O_3_/g-C_3_N_4_ composites for the cataluminescence sensing of H_2_S [30]. An efficient dielectric barrier discharge (DBD) plasma-assisted method for the fabrication of the g-C_3_N_4_-Mn_3_O_4_ composite was investigated by Hu et al., which displayed a highly selective, sensitive, and linear cataluminescence (CTL) response towards H_2_S gas [31]. Sanjay Mathur et al. synthesized SnO_2_ nanowires via the CVD method, and the SnO_2_ nanowires exhibited an excellent photoresponse performance [32]. Kuang et al. have synthesized high-yield SnO_2_ nanowires via an Au catalytic vapor-liquid-solid (VLS) growth process and the SnO_2_ nanowire-based humidity sensor displayed a fast response and high sensitivity to relative humidity changes at room temperature [33]. The three-dimensional network’s SnO_2_ nanowire was prepared via a flame-based thermal oxidation process (FTS) and applied for ethanol sensing [34]. Oleg Lupan et al. investigated the hybrid networks of heterogeneous shell-core Ga_2_O_3_/GaN:O_x_@SnO_2_ nano- and micro-cables with a shell in mixed phases for improving sensor properties [35]. However, to our best knowledge, there is still no research focused on the design and gas sensing application of the g-C_3_N_4_ nanosheets-decorated SnO_2_ flower-like nanorods.

Herein, the hydrothermal method was utilized for the first time to synthesis the g-C_3_N_4_ nanosheets-decorated SnO_2_ flower-like nanorods for the ethanol sensing application. It was found that the g-C_3_N_4_ nanosheets-decorated tin oxide flower-like nanorods composite possesses a much higher response value, repeatability, and stability to ethanol vapor than pure flower-like SnO_2_ nanorods.

## 2. Results and Discussion

### 2.1. Sample Characterization

**Figure 1:**
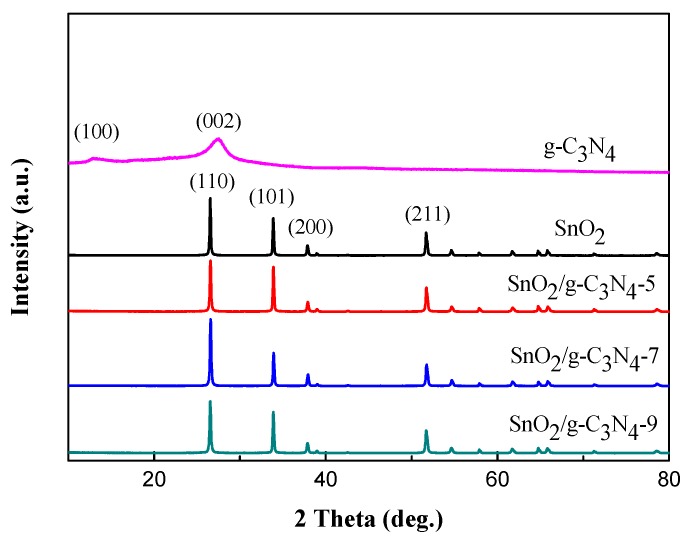
X-ray powder diffraction (XRD) patterns of the synthesized SnO_2_, g-C_3_N_4_, and g-C_3_N_4_nanosheets-decorated tin oxide flower-like nanorods composites (SnO_2_/g-C_3_N_4_-5, SnO_2_/g-C_3_N_4_-7, and SnO_2_/g-C_3_N_4_-9).

The pure SnO_2_ and SnO_2_/g-C_3_N_4_ composites with 5, 7, and 9 wt % g-C_3_N_4_ contents were synthesized by a facile hydrothermal method. Also, the as-prepared samples were marked as SnO_2_/g-C_3_N_4_-5, SnO_2_/g-C_3_N_4_-7, and SnO_2_/g-C_3_N_4_-9, respectively. Figure 1 displays the XRD patterns of the synthesized SnO_2_, g-C_3_N_4_, and g-C_3_N_4_nanosheets-decorated tin oxide flower-like nanorods (SnO_2_/g-C_3_N_4_) composites with different g-C_3_N_4_ contents. One can see from the XRD pattern that two diffraction peaks at 13.1° and 27.5° can be observed for pure g-C_3_N_4;_these two peaks were accorded to the (100) plane and (002) plane of g-C_3_N_4_,which could be due to the inter-layer structure of the tri-s-triazine unit with interplannar spacing and the conjugated aromatic system, respectively [36]. The XRD patterns of SnO_2_/g-C_3_N_4_ composites show some diffraction peaks at 26.61°, 33.89°, 37.94°, and 51.78°, which could be assigned to the (110), (101), (200), and (211) planes of the tetragonal rutile structure SnO_2_ (JCPDS Card No. 41-1445). However, the diffraction peaks of g-C_3_N_4_ are not observed in the SnO_2_/g-C_3_N_4_ composites. This could be due to the small content of g-C_3_N_4_.

**Figure 2:**
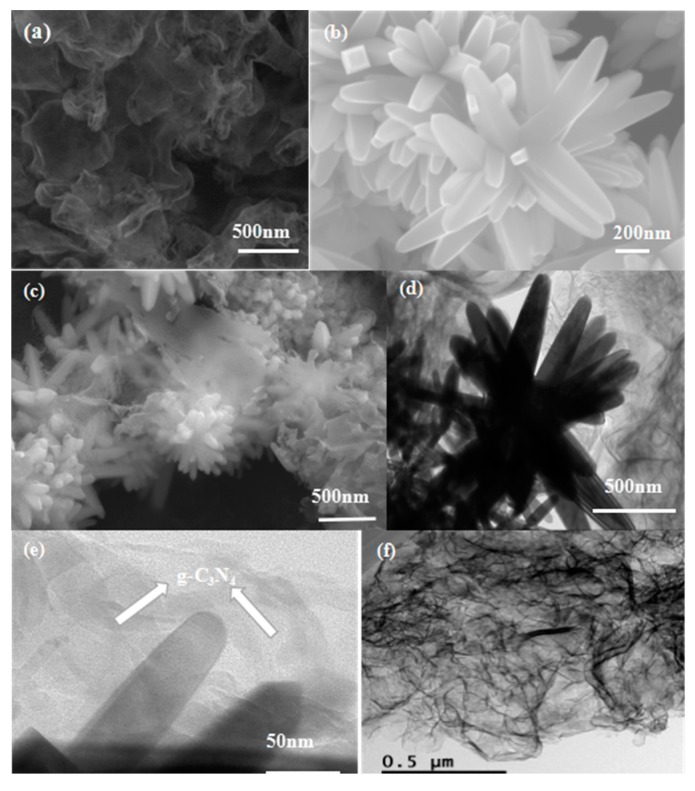
Field-emission scanning electron microscopy (FESEM) images of pure g-C_3_N_4_ (**a**); SnO_2_ flower-like nanorods (**b**); SnO_2_/g-C_3_N_4_nanocomposite (**c**); and transmission electron microscopy (TEM) images of the SnO_2_/g-C_3_N_4_ composite (**d**,**e**) and pure g-C_3_N_4_ (**f**).

The microstructure and morphology of the synthesized samples were verified by using FESEM and TEM. One can see clearly from Figure 2a that the morphology of the as-prepared g-C_3_N_4_ possesses many wrinkles with overlaps at the edges, demonstrating the existence of the two dimensional (2D) nano-lamellar structure. It can be observed from Figure 2b that the pure SnO_2_ product displays the unique 3D flower-like microstructure assembled with many uniform nanorods. The lengths and diameters of a single nanorod are about 400–600 nm and 50–100 nm, respectively. For the g-C_3_N_4_ nanosheets-decorated SnO_2_ flower-like nanorods composite, as shown in Figure 2c, the SnO_2_ flower-like nanorods were closely coupled by g-C_3_N_4_ nanosheets. A proposed growth mechanism of SnO_2_ flower-like nanorods can be summarized by crystal growth and nucleation theory. The SnO_2_nanocrystals nucleation points are generated in different orientations. Therefore, the SnO_2_ nanorods grow in irregular directions and finally formed into 3D flower-like structures. Figure 2f displays the typical TEM image of pure g-C_3_N_4_, and g-C_3_N_4_possesses two dimensional nanosheets structure with many wrinkles. The TEM images of g-C_3_N_4_ nanosheets-decorated tin oxide flower-like nanorods composites are displayed in Figure 2d,e, and SnO_2_ flower-like nanorods were coupled by a lamellar structure, which is 2D g-C_3_N_4_. Thus, we can conclude that g-C_3_N_4_ nanosheets-decorated SnO_2_ flower-like nanorods composites were successfully synthesized by the hydrothermal method combining the above analysis results offered by XRD, FESEM, and TEM.

**Figure 3:**
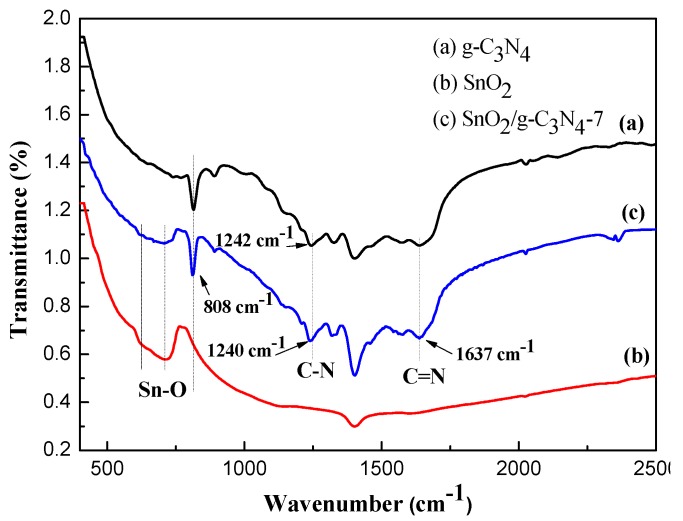
Fourier transform infrared spectrometer (FT-IR) spectra of g-C_3_N_4_ (**a**); SnO_2_ (**b**); and SnO_2_/g-C_3_N_4_-7 (**c**) nanocomposite.

Figure 3 exhibits the FT-IR spectra of g-C_3_N_4_, SnO_2_, and SnO_2_/g-C_3_N_4_-7 samples. As can be seen from Figure 3b, the broad absorption peaks could be observed at wave-numbers of 570 cm^−1^ and 660 cm^−1^, which could be assigned to the Sn–O characteristic peaks. In Figure 3a,c, the peaks in the range of 1240–1637 cm^−1^ are ascribed to the C–N and C=N stretching vibration modes, and the peak at 808 cm^−1^ corresponds to the triazine units. These two sets of characteristic vibration peaks are characteristic of g-C_3_N_4_. As is shown in Figure 3c, all the characteristic peaks of SnO_2_ and g-C_3_N_4_ can be observed clearly. These results make up for our XRD analysis, in which g-C_3_N_4_ and SnO_2_ are coexisting in the SnO_2_/g-C_3_N_4_ composites. Compared with pure g-C_3_N_4_, there is a slight red shift in the bands of g-C_3_N_4_ in the composite. This result indicates that there is an interaction between SnO_2_ and g-C_3_N_4_ [27], which is beneficial to the gas sensing application.

**Figure 4:**
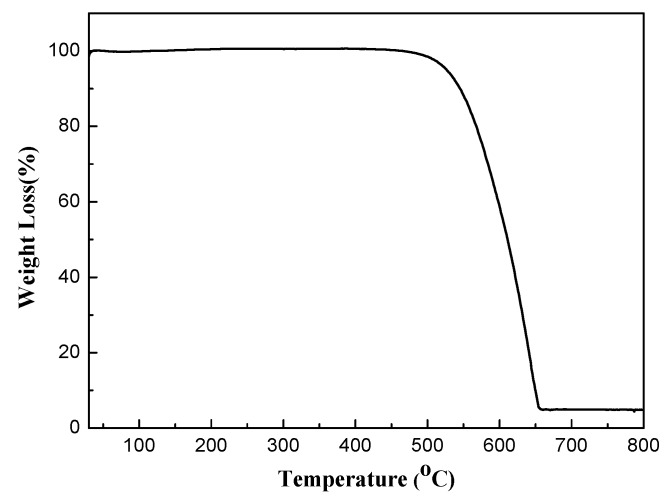
Thermogravimetry (TG) analysis for heating the g-C_3_N_4_from room temperature to 800 °C.

TG analysis was investigated by heating up from room temperature to 800 °C at a heating rate of 5 °C·min^−1^ to reveal the weight change of g-C_3_N_4_. It can be seen from Figure 4 that the weight of g-C_3_N_4_ is set constant at temperature below 500 °C. When the temperature increases to 510 °C, the weight of g-C_3_N_4_ starts to decrease (the combustion of g-C_3_N_4_ in air). The weight stays at the same level when the temperature exceeds 655 °C. It can be concluded that g-C_3_N_4_ is stable at low temperature and burn at high temperature. This phenomenon demonstrates that g-C_3_N_4_ could stably exist in the composite under the operating temperature in the range of 200–400 °C in the gas-sensing test process.

### 2.2. Sensing Performance Tests

**Figure 5:**
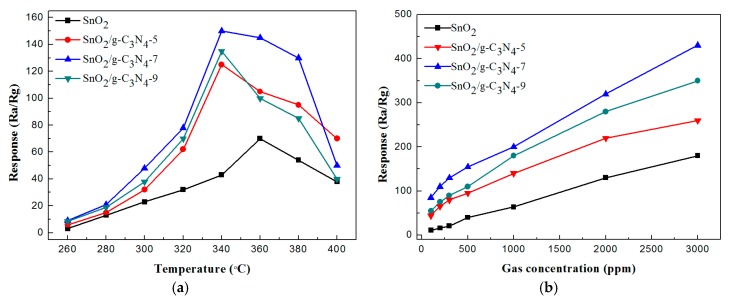
The response values of the SnO_2_, SnO_2_/g-C_3_N_4_-5, SnO_2_/g-C_3_N_4_-7, and SnO_2_/g-C_3_N_4_-9 to 500 ppm ethanol (**a**) under different operating temperatures; (**b**) for different concentrations of ethanol at 340 °C.

In order to investigate the gas sensing performance of the synthesized samples-based sensor to ethanol, a series of tests were performed. The response values (*R_a_*/*R_g_*) of the g-C_3_N_4_nanosheets-decorated tin oxide flower-like nanorods composite and the pure flower-like SnO_2_-based gas sensors toward 500 ppm ethanol vapor were measured under different operating temperature. With the increase of operating temperature, one can see from Figure 5a that all of the samples exhibited the similar variation tendency. Also, the response values of the SnO_2_/g-C_3_N_4_-based sensors reached the maximum value at 340 °C, while the maximum value of pure SnO_2_ was 70 at 360 °C. This result shows that the optimum operating temperature of SnO_2_/g-C_3_N_4_ decreased compared with that of pure SnO_2_. This result may be due to the fact that the chemisorbed oxygen species can achieve the required energy and effectively react with ethanol vapor molecules on sample surface varying the resistance significantly [37]. The response value of the SnO_2_/g-C_3_N_4_ composite-based sensor is much higher than pure SnO_2_. The response values increased with adding the content of g-C_3_N_4_ from 5 wt. % to 7 wt. % and decreased with further increase of g-C_3_N_4_ content. The response values of the pure SnO_2_, SnO_2_/g-C_3_N_4_ composites with g-C_3_N_4_ contents of 5, 7, and 9 wt. % to 500 ppm ethanol vapor at 340 °C are 43, 125, 150, and 135, respectively, indicating that the addition of g-C_3_N_4_ has a great influence on enhancing the gas sensing performance. When the mass percentage of g-C_3_N_4_ in the composites is 7 wt. %, the response reaches the maximum value. A suitable content of g-C_3_N_4_ in the composite is beneficial to form the preferable heterojunction structure in the interface region between flower-like nanorods SnO_2_ and 2D g-C_3_N_4_. However, much higher addition of g-C_3_N_4_may result in the formation of the connection of bulk. This will further decrease the specific surface area of the sample and reduce the active sites for oxygen and ethanol gas adsorption, further leading to the degradation of sensing performance. Hence, the optimum operating temperature is 340 °C and the optimum g-C_3_N_4_ content is 7 wt. % for this g-C_3_N_4_ nanosheets-decorated SnO_2_ flower-like nanorods nanomaterial. Therefore, all of the further research was carried out by using SnO_2_/g-C_3_N_4_-7 composite sensor at 340 °C. Figure 5b displays the response values of the four samples to different ethanol vapors in the concentration range of 100–3000 ppm at 340°C. With the increase of ethanol concentration, the response values increased for all of these four sensors. The curves increased rapidly in the range of 100–500 ppm and increased slowly with the increasing concentrations from 500 ppm to 3000 ppm.

**Figure 6:**
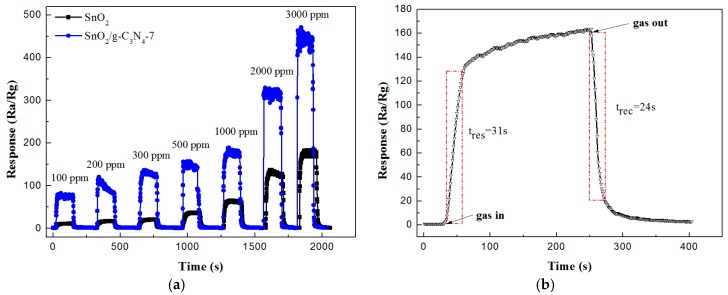
(**a**) The real time response curves of the SnO_2_ and SnO_2_/g-C_3_N_4_-7 composite sensors toward ethanol vapor; (**b**) response-recovery time characteristics of the SnO_2_/g-C_3_N_4_-7 based sensor to 500 ppm ethanol vapor at 340 °C.

Figure 6a shows the real time response curves of the pure SnO_2_ and SnO_2_/g-C_3_N_4_-7 to ethanol in the range of 100–3000 ppm at 340 °C. All of the response-recovery cycles were measured about 300 s with a response interval and a recovery interval of 150 s, respectively. We can observe from Figure 6a that the two samples show a similar trend: the response values increase with increasing ethanol concentration. To the same concentration of ethanol, SnO_2_/g-C_3_N_4_-7 exhibited much higher response value than that of pure SnO_2_. The response value of the SnO_2_/g-C_3_N_4_-7 composite-based sensor towards 500 ppm ethanol vapor was about 150, about four times higher than pure SnO_2_. As is known, response-recovery time is another very important influential factor on the application of gas sensor. Figure 6b shows the response-recovery time curve of the SnO_2_/g-C_3_N_4_-7 composite toward 500 ppm ethanol vapor. As seen from the curve, when the sensor was exposed and separated to ethanol, the response increased rapidly (31 s) and also decreased rapidly (24 s), respectively.

**Figure 7:**
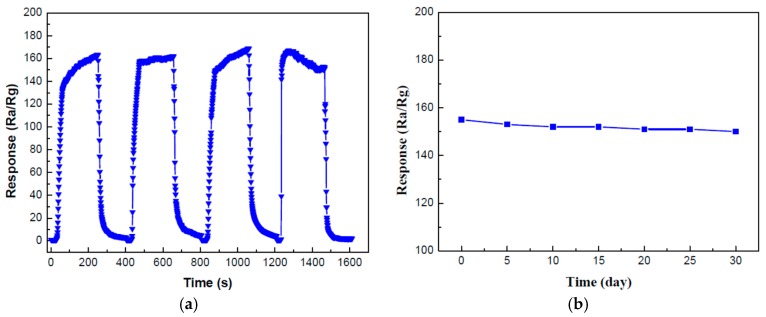
(**a**) Repeatability and (**b**) stability measurements of the SnO_2_/g-C_3_N_4_-7-based sensor to 500 ppm ethanol at 340 °C.

The repeatability and stability are both crucial influence factors of gas sensing performances. As is shown in Figure 7a, the response values of these four response-recovery cycles of SnO_2_/g-C_3_N_4_-7 sensor stay almost the same (165, 160, 167, and 155) toward 500 ppm ethanol at 340 °C. This result indicated that the synthesized SnO_2_/g-C_3_N_4_-7 composite possesses an admirable repeatability for ethanol vapor detection. Figure 7b displays the stability test result of SnO_2_/g-C_3_N_4_-7 composite sensor toward 500 ppm ethanol vapor at 340 °C, and the response values were kept at a stability of around 155 after 30 days test, indicating that the synthesized g-C_3_N_4_ nanosheets-decorated SnO_2_ flower-like nanorods composite possesses an excellent stability.

**Figure 8:**
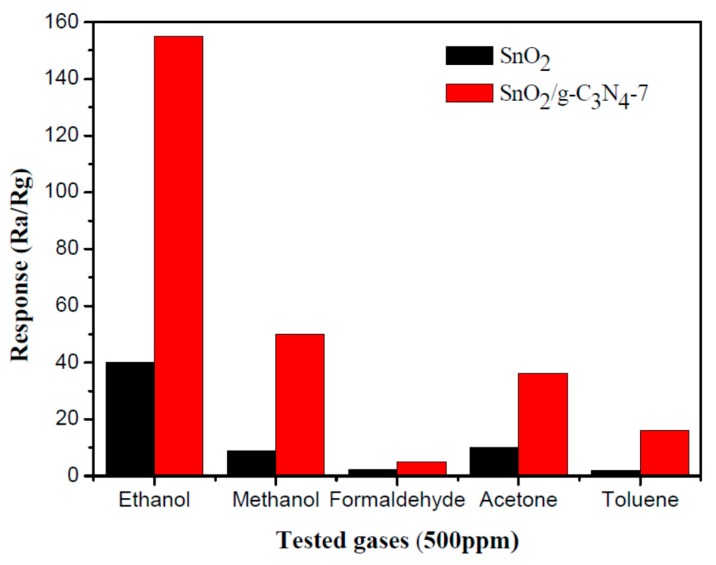
Comparision of the response values of the flower-like SnO_2_ nanorods and the SnO_2_/g-C_3_N_4_-7 composite toward 500 ppm different gases at 340 °C.

As is well known, the selectivity of the sensor for the different gases is one of the most important factors for its practical application. Figure 8 displays the selectivity test results of SnO_2_ flower-like nanorods and SnO_2_/g-C_3_N_4_-7 composite to methanol, ethanol, toluene, formaldehyde, and acetone with the concentration of 500 ppm. The test results indicated that the sample possesses the superior selectivity to ethanol vapor at the operating temperature of 340 °C. The high selectivity to ethanol maybe come from the fact that when reacted with the absorbed oxygen, ethanol is more likely to lose electrons, and hydroxyl group (–OH) is easy to oxidize under the optimum operating condition.

Table 1 lists the sensing performances of different materials to ethanol vapor. One can observe from Table 1 that the RGO/hollow SnO_2_ nanoparticles [11], hollow ZnO/SnO_2_ spheres [16], tubular α-Fe_2_O_3_/g-C_3_N_4_ [29], and Au/3D SnO_2_ microstructure [38] samples possess the response values to ethanol of 70.4, 78.2, 7.76, and 30, respectively. In our research, the 7 wt. % 2D g-C_3_N_4_ decorated SnO_2_ flower-like nanorods composite possesses the response value of 85 to 100 ppm ethanol vapor at 340 °C, indicating a great potential application of the synthesized sample to ethanol detection.

### 2.3. Mechanism Discussion

**Figure 9:**
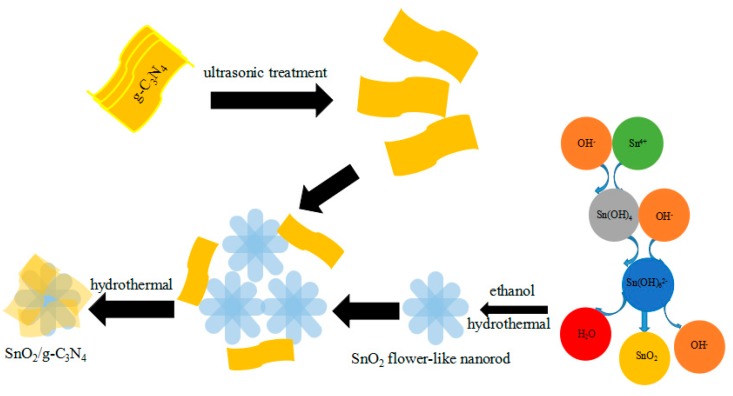
The schematic diagram of the synthesis of the g-C_3_N_4_ nanosheets decorated SnO_2_ flower-like nanorods.

SnO_2_ flower-like nanorods have been synthesized via a hydrothermal reaction method, and the schematic diagram of the synthesis of the g-C_3_N_4_ nanosheets-decorated SnO_2_ flower-like nanorods is displayed in Figure 9. Many researchers hold the point that the diameter of the SnO_2_ nanorods is changed by varying the Sn^+^/OH^−^ ratio in solution [39]. However, Vuong et al. declared that the diameter of SnO_2_ nanorods decreased with the increase of the stannic chloride amount. A proposed growth mechanism of SnO_2_ flower-like nanorods can be summarized by crystal growth and nucleation theory. The synthesis process includes two sections of nucleation and crystal growth. In the hydrothermal condition, the Sn(OH)_6_^2−^ nucleus is formed via the following chemical reactions in the process of nucleation stage [40]:

(1) $${\mathrm{Sn}}^{4+}+4{\mathrm{OH}}^{-}↔\mathrm{Sn}{\left(\mathrm{OH}\right)}_{4}$$

(2) $$\mathrm{Sn}{\left(\mathrm{OH}\right)}_{4}+2{\mathrm{OH}}^{-}↔\mathrm{Sn}{\left(\mathrm{OH}\right)}_{6}^{2-}$$

(3) $$\mathrm{Sn}{\left(\mathrm{OH}\right)}_{6}^{2-}\to {\mathrm{SnO}}_{2}+H_{2}O+2{\mathrm{OH}}^{-}$$

In the process, nucleation plays an important role not merely in the morphology formation but also in the quantity of the final product. At the initial stage of the chemical reaction, the Sn^4+^ ions start to react with the redundant OH^−^ ions and further form the [Sn(OH)_6_]^2−^ coordination ions. Meanwhile, a small quantity of SnO_2_nanocrystals can be generated due to the decomposition of the [Sn(OH)_6_]^2−^ coordination ions. These initial SnO_2_nanocrystals play an important role as seeds in the next hydrothermal stage. The [Sn(OH)_6_]^2−^ coordination ions could be accelerated to decompose and form plenty of SnO_2_nanocrystals in the later hydrothermal reaction condition, which can be aggregated into SnO_2_ nanoparticles. At the same time, these SnO_2_ nanocrystals nucleation points around [Sn(OH)_6_]^2−^ can be oriented to grow into the rod-like structures. The SnO_2_ nanocrystals nucleation points are generated in different orientations. As a result, the SnO_2_ nanorods grow in irregular directions and finally form into 3D flower-like structures. This phenomenon can be explained by the fact that the surface-free energy of the rutile structure of crystalline SnO_2_ faced an increase in the order of (110) < (100) < (101) < (001), which lead to the crystal growth on the faces of (001) or (101). However, the other faces have no exceptions [41,42].

**Figure 10:**
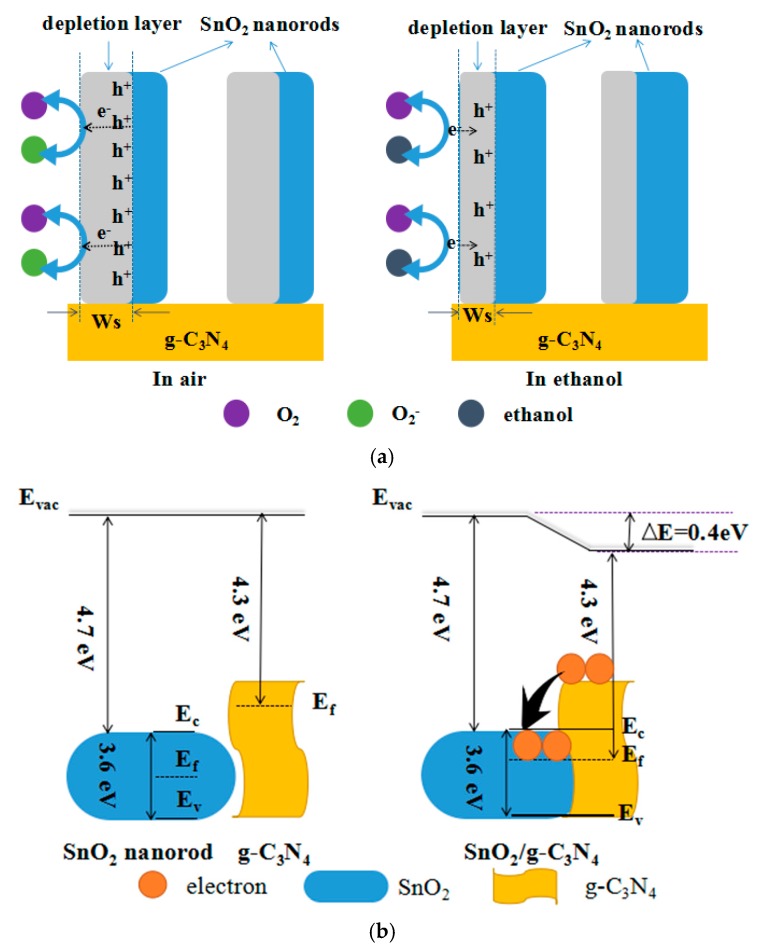
(**a**) The schematic diagram of air and ethanol react with the synthesized composite; and (**b**) the band diagram of SnO_2_/g-C_3_N_4_ before and after the combination.

2D g-C_3_N_4_ nanosheets-decorated SnO_2_ flower-like nanorods composites were synthesized via a facile hydrothermal method as a high-property gas sensor for detecting ethanol. In order to understand the gas-sensing process, the schematic diagram of the test gas that reacted with SnO_2_/g-C_3_N_4_ composite was shown in Figure 10a. As is known, the similar principle of gas sensor is the surface-adsorbed oxygen theory. When the sensor was exposed in the air condition, the oxygen molecules were adsorbed on the SnO_2_ surface and capture electrons from the conduction band of SnO_2_. Furthermore, the adsorbed oxygen molecules were ionized into O^2−^, O^−^, and O_2_^−^ (Equation (4)), and formed a depletion layer with a certain width (W_s_) of the hole accumulation (h^+^) on the SnO_2_ surface [43,44]. When the sensor was exposed in ethanol gas, the reduced gas ethanol molecules were oxidized into acetaldehyde and finally turned into carbon dioxide and water by these oxygen anions (Equations (5) and (6)) [10]. As a result, the trapped electrons were released back to the SnO_2_, depletion layer, where the width (W_s_) of depletion area of the hole accumulation (h^+^) became thinner and led to the decrease of the resistance by the transfer of electrons between ethanol molecules and oxygen anions. As is well known, the electrons transfer may affect the great change of the composite resistance. In addition, the improved ethanol-sensing performances of the g-C_3_N_4_ nanosheets-decorated flower-like SnO_2_ nanorods composite could be attributed to the structure of SnO_2_ nanorods coupled by 2D g-C_3_N_4_nanosheets and the heterojunction of interface region between flower-like SnO_2_ nanorods and 2D g-C_3_N_4_.

(4) $$O_{2}+e^{-}\to O_{2}^{-}$$

(5) $$2{\mathrm{CH}}_{3}{\mathrm{CH}}_{2}\mathrm{OH}+O_{2}^{-}\to 2{\mathrm{CH}}_{3}\mathrm{CHO}+2H_{2}O+e^{-}$$

(6) $$2{\mathrm{CH}}_{3}\mathrm{CHO}+5O_{2}^{-}\to 4{\mathrm{CO}}_{2}+4H_{2}O+5e^{-}$$

In general, the large specific area of 2D g-C_3_N_4_ nanosheets can provide more active sites to adsorb oxygen molecules and ethanol molecules. The interconnecting network structure created by SnO_2_ nanorods and 2D g-C_3_N_4_ nanosheets could supply more channels for the gas adsorption and diffusion and further enhance the interaction between SnO_2_ and ethanol molecules. The energy band model (Figure 10b) was used to explain the energy change of SnO_2_/g-C_3_N_4_ for ethanol detection. Figure 10b shows that the g-C_3_N_4_ and SnO_2_ have the structure of valence band and conduction band (E_v_ and E_c_) and the Fermi level (E_f_) is between these two bands. When flower-like SnO_2_ nanorods and 2D g-C_3_N_4_ nanosheetswere combined together, a heterojunction structure was formed. When ethanol molecules pass through the interface between g-C_3_N_4_ and SnO_2_, the electrical property at the heterojunction was changed. SnO_2_ and g-C_3_N_4_ are all n-type semiconductors with band gaps of 3.6 eV and 2.7 eV, respectively. Since the work function of g-C_3_N_4_ is smaller than that of SnO_2_, the electrons will inflow from the conduction band of g-C_3_N_4_ to the conduction band of SnO_2_, leading to a higher potential barrier. The fermi level is aligned when the electronic transmission achieves a new dynamic balance. The electrons may go over the low energy barriers and the schottky barrier is 0.4 eV. As a result, the electrons and holes are separated [27,43]. Meanwhile, the heterojunction structure may suppress the recombination of electron-hole and urge electrons to transfer quickly from ethanol vapour to the surface of SnO_2_/g-C_3_N_4_. Therefore, this leads to a higher response because of the increased conductivity of the heterojunction structure [29].

## 3. Materials and Methods

### 3.1. Chemicals

Analytical-grade purity SnCl_4_·5H_2_O (99.0%), NaOH, and absolute ethyl alcohol were purchased from Shanghai Macklin Biochemical Co., Ltd, Shanghai, China and were used without further purification.

### 3.2. Sample Preparation

Graphitic carbon nitride (g-C_3_N_4_) was synthesized by our previous reported method [45]. Typically, 7 wt. % g-C_3_N_4_ nanosheets-decorated SnO_2_ flower-like nanorods (SnO_2_/g-C_3_N_4_-7) were synthesized by the hydrothermal method: 0.17 g g-C_3_N_4_ powder was dispersed into 200 mL ethanol under ultrasonic treatment for 2 h. 5.259 g SnCl_4_·5H_2_O was added into 200 mL of NaOH solution (0.81 M). Subsequently, the g-C_3_N_4_ solution was added into this mixture solution with magnetic stirring until it formed a white suspension. Finally, the mixture was transferred into a 500 mL stainless-steel Teflon-lined autoclave, then put into an oven and further heated at 200 °C for 48 h. The final product was washed with deionized water and ethanol several times and dried at 60 °C. According to this method, the SnO_2_/g-C_3_N_4_ composites with 5 and 9 wt. % g-C_3_N_4_ content were also synthesized and marked as SnO_2_/g-C_3_N_4_-5 and SnO_2_/g-C_3_N_4_-9, respectively. The pure flower-like SnO_2_nanorods were also synthesized by the same method.

### 3.3. Characterizations

X-ray diffraction (XRD) analysis was carried out on Bruker-AXS D8 (Bruker, Madison, WI, USA) with CuKα radiation at 40 kV and 25 mA. Fourier Transform Infrared Spectrometer (FT-IR) was recorded on a Bruker Tensor 27 (Bruker, Madison, WI, USA). Thermogravimetry (TG) analysis was completed on a NETZSCH STA449C Simultaneous Thermal Analyzer (NETZSCH, Selb, Germany) at a heating rate of 10 °C·min^−1^ under air atmosphere. Field-emission scanning electron microscopy (FESEM) (Quanta™ 250 FEG, FEI, Eindhoven, The Netherlands) was used to observe the structure and morphology of the sample. Transmission electron microscopy (TEM) analysis was done on a JEOL JEM-2100 microscope (JEOL, Tokyo, Japan) operating at 200 kV.

### 3.4. Gas Sensor Fabrication and Analysis

**Figure 11:**
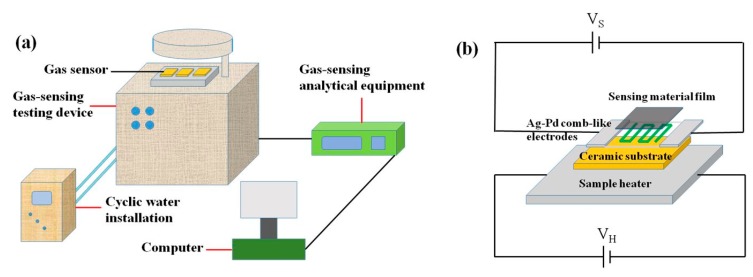
(**a**) The schematic diagram of the CGS-4TPS gas-sensing test system and (**b**) the gas sensor structure.

Gas-sensing performance test of the synthesized sample was carried out on an intelligent gas-sensing analysis system of CGS-4TPS (Beijing Elite Tech. Co., Ltd., Beijing, China). Figure 11 shows the schematic diagram of the system, the sensor structure, and the working principle. In the sensor fabricate process, the synthesized sample was mixed with several drops of distilled water to form a paste. Then, a ceramic substrate (13.4 mm × 7 mm, screen-printed with Ag-Pd comb-like electrodes) was coated onto the paste to obtain the resistance-type sensor. Before the gas sensing test, the sensor was aged at 200 °C for 12 h to improve its stability and repeatability. In the sensing performance test process, the test gas was first injected into the closed 0.018 m^3^ volume chamber by a microinjector with the relative humidity of 40%. The operating temperature was set in the range of 200 °C to 400 °C. The gas response (S) was defined as the ratio of *Ra*/*Rg*, where *Ra* and *Rg* were the resistances of sensor in air and in the test gas, respectively. The response and recovery times were defined as the time required for a change in response to reach 90% of the equilibrium value.

## 4. Conclusions

In summary, the g-C_3_N_4_ nanosheets-decorated tin oxide flower-like nanorods (SnO_2_/g-C_3_N_4_) composite was successfully synthesized by using a facile hydrothermal method. The as-prepared sample possesses flower-like nanorods and a lamellar structure. Compared with pure SnO_2_, the g-C_3_N_4_ nanosheets-decorated SnO_2_ flower-like nanorods exhibited an obvious improvement of gas sensing performance to ethanol, and the response value was 150 to 500 ppm ethanol at 340 °C. The improved sensing properties are mainly attributed to the high surface area of the sample and the heterojunction between SnO_2_ and g-C_3_N_4_. Considering the effective synthesis approach and the high sensing performance, the as-prepared SnO_2_/g-C_3_N_4_ composite could be an ideal candidate for ethanol detection application.

## Figures and Tables

**Table 1:** The ethanol sensing performance of the previous reported results and this work.

Materials	Ethanol Vapor Concentration (ppm)	Temperature (°C)	Response (*R_a_*/*R_g_*)	Ref.
RGO/hollow SnO_2_	100	300	70.4	[11]
Hollow ZnO/SnO_2_ spheres	100	225	78.2	[16]
α-Fe_2_O_3_/g-C_3_N_4_	100	340	7.76	[29]
Au/3D SnO_2_ microstructure	150	340	30	[38]
SnO_2_/g-C_3_N_4_	100	340	85	this work
